# The Role of Congenital Heart Disease Patient Organizations in Advocacy, Resources, and Support Across the Lifespan

**DOI:** 10.1016/j.cjcpc.2023.08.006

**Published:** 2023-09-12

**Authors:** Shelagh Ross, Amy Verstappen

**Affiliations:** a Global Alliance for Rheumatic & Congenital Hearts (Global ARCH), Victoria, British Columbia, Canada; b Global Alliance for Rheumatic & Congenital Hearts (Global ARCH), Philadelphia, Pennsylvania, USA

## Abstract

Congenital heart disease patient organizations, comprising experts with lived experience, and their families and supporters, have become an essential voice for patient advocacy, resources, and support. Thanks largely to the Internet, these organizations are growing in number worldwide. Their common voice can be used to influence research, be the catalyst for advocacy efforts for new programmes and supports, and connect patients and providers in endeavours beyond the clinical setting. The result has become more active engagement with how policy decisions, research directions, and laws are decided that will shape patients’ lives. From advocating for much-needed mental health support, policies to combat discrimination and the lack of access to support services, and partnerships with clinicians and others to develop educational resources and tools, congenital heart disease patient organizations are having a considerable impact on patient lives and ultimately patient outcomes.

## Graphical abstract

**Figure:**
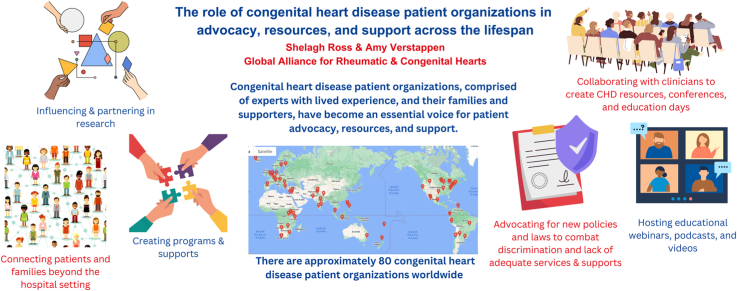

## Résumé

Les organisations formées de patients atteints d’une cardiopathie congénitale (CC), qui sont de par leur vécu des experts en la matière, de membres de leur famille et de personnes qui les soutiennent, jouent un rôle crucial pour défendre les intérêts des patients et leur offrir des ressources et du soutien. Dans une large mesure grâce à l’Internet, le nombre de ces organisations est en croissance partout au monde. La mise en commun de leur voix peut influencer la recherche, servir de catalyseur aux initiatives en faveur de nouveaux programmes et de nouvelles formes de soutien, et contribuer à établir des liens entre les patients et les cliniciens au-delà du contexte clinique. La prise de décisions concernant les politiques, les orientations de recherche et les lois peut par conséquent bénéficier d’une participation plus active des patients, qui sont les premiers concernés. Ces organisations exercent une grande influence sur la vie des patients et, en fin de compte, sur leurs résultats cliniques, notamment grâce à tous les efforts qu’ils consacrent à la revendication d’un soutien en santé mentale, qui fait grandement défaut, de politiques de lutte contre la discrimination et le manque d’accès aux services de soutien ainsi que de partenariats avec des cliniciens et d’autres alliés dans le but de concevoir des ressources et des outils éducatifs.

**The** **R** **ole of** **P** **atien** **t** **O** **rganizations** **in** **S** **upport,** **R** **esources, and** **A** **dvocacy for** **P** **atients** **W** **ith** **C** **ongenital** **H** **eart** **D** **isease** **A** **cross the** **L** **ifespan**

**“My heart condition affects where I choose to live, where I choose to work, what I choose to study, where I feel comfortable vacationing, if I’ll have children. It affects everything.”**^1^

Every parent of a child born with congenital heart disease (CHD) wants the best possible life for their child, and every person living with CHD wants to thrive despite their condition. Over the last 20 years there has been tremendous progress in CHD research and treatment, but major gaps in care continue that put patient lives and well-being at risk. Many patients and families continue to perceive postsurgical CHD as “fixed” and lack sufficient information and skills to best optimize their lifelong heart health. The time demands of managing complex medical needs can result in employment challenges and loss of income,^1^ and adults with CHD can face workplace discrimination and challenges accessing needed insurance and benefits.^2^ Finally, research shows that high numbers of CHD parents and adult patients with CHD (ACHD) struggle with anxiety and depression,^3^^,^^4^ and yet there is a severe lack of mental health services and social support available to the community with CHD. Patient organizations^2^ are essential to improving the lives of patients and their families and are uniquely positioned to address real-world issues that limit patient health and well-being.

## Empowering the Collective Patient Voice

In 2021, the World Health Organization released “Nothing For Us, Without Us,” which states that people living with disease are “experts in their own right” and must be “key partners and drivers in the cocreation, implementation, and evaluation” of all policies, programmes, and services that serve their population.^5^ The framework emphasizes that meaningful engagement must go beyond soliciting patient input to a redistribution of power from clinicians, researchers, and policy makers to the patients themselves. Patient organizations have a key role to play in bringing together individuals living with disease to define common needs and take collective action on behalf of their community.

This collective action is what defines advocacy and can be described in 3 levels: self-advocacy, collective advocacy, and systems advocacy.^6^ *Self-advocacy* is empowering an individual to identify their own needs and speak up for themselves. *Collective advocacy* focuses on creating a common voice on behalf of a particular group. For many individuals, being part of a CHD group is essential to self-empowerment, as it allows patients and families to overcome the fear, shame, and stigma often associated with CHD. It is also essential in recognizing not just one’s individual needs, but the common needs of the larger community. This is especially important in CHD, which involves a heterogeneous population, varying widely in severity, comorbidities, and treatments, resulting in patients whose experiences are perhaps uniquely diverse.

Virtually, all patient organizations engage in both self-advocacy and collective advocacy, and as such serve as advocacy organizations. Once these collective needs are defined, many also tackle *systems advocacy*, which involves actions aimed at changing policies, laws, or rules in order to enhance health or well-being. Many of these efforts have resulted in major successes in improving access to the high-quality care, lifelong care, and social support essential for ensuring that every person with CHD can thrive.

## Availability of Patient Organizations

**Figure 1:**
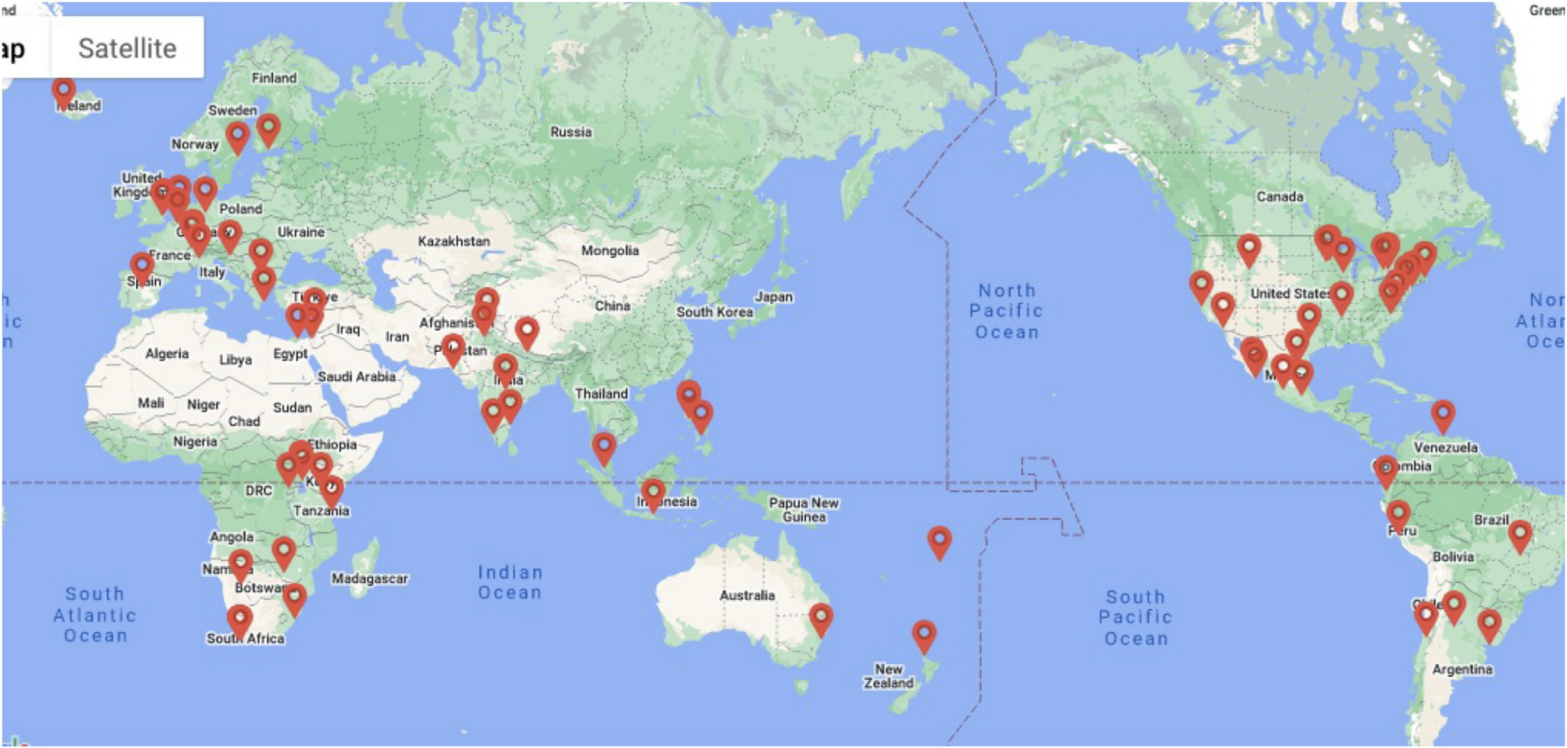
Global Alliance for Rheumatic & Congenital Hearts (Global ARCH) map of member organizations. Used with permission from Global ARCH.

Currently, there are over 80 patient organizations serving patients with CHD around the world, and close to 60 from 40 countries belong to the Global Alliance for Rheumatic & Congenital Hearts (Global ARCH),^7^ an organization whose mission is to unite and empower congenital and rheumatic heart disease patient organizations to improve global outcomes in childhood-onset heart disease (Fig. 1).

Over half of these organizations are in low-resource settings in which neither surgical services nor adequate assistance with the costs of CHD care is typically available. The primary focus of these groups is often helping families raise funds for care, which is unnecessary in most high-income countries. However, activities such as social and emotional support, education, and advocacy are common worldwide.

## Limitations of Patient Organizations

Selection bias is an ongoing challenge that organizations must strive to overcome if they want to serve the broader population of patients and families living with CHD. By their very nature, these organizations tend to attract the sickest patients and those navigating procedures, who are most likely to be looking for information, connection, and support. Those who are feeling well, or whose child is doing well, tend not to be as involved. Patients who perceive their CHD to be “cured” will be unlikely to seek out either patient groups or clinical care. Although lower levels of education and income, and being a member of a racial minority, have a major negative impact on CHD morbidity and mortality,^8^ many patient organizations in high-income countries lack participation by racial minorities and are dominated by the more affluent and educated. Educational information available is typically written for the college educated, whereas the average person in Canada and many other high-income countries reads below the high school level.^9^ At minimum, group participation requires leisure time, access to a computer, language facility, and good Internet access, which can be a challenge for many disadvantaged communities.

CHD groups around the world increasingly recognize that to address the needs of the most vulnerable, they must be more inclusive, and are now making specific efforts to reach these communities. This includes creating accessible information and materials, easy-to-navigate websites, and making sure that their online and in-person events include people from different cultures, backgrounds, and abilities.

## Clinical Settings vs the “Real World”

It is important to recognize that, by definition, health care professionals interact with patients and families in a clinical setting. They will only see those still engaged in care, and clinic interactions will focus predominantly on medical issues, with the total interaction often consisting of less than 30 minutes every 6 months to 2 years. Patients and families are more likely to make and keep cardiology appointments when they are experiencing active health problems or are facing major care decisions or procedures. In contrast, patient organizations regularly interact with patients and caregivers online, at social events, and in their community. The conversations will focus not just on the medical challenges of their disease but include the “whole world” of CHD—its impact on work, family life, finances, and their own fears and hopes. Even people who perceive CHD as “cured” may continue to engage in Facebook groups and follow Instagram accounts and maintain their connection to the community with CHD.

This broader view allows patient organizations to identify unmet needs that clinicians may not see, or whose impact they do not appreciate. They can raise awareness of these issues, provide services that fill these gaps in novel ways, and partner with researchers and clinicians to ensure that these issues are recognized and addressed. They can also engage directly with policy makers to ensure that their rights to health and well-being are met. In this article, we have chosen to highlight 3 areas of activity that are a major focus of many patients and family organizations: support, resources, and advocacy.

## Promoting Mental Health Through Social Support

“I think growing up one of the hardest things for me was not knowing anyone like me. I wish my doctors would have put me and my family in touch with other families like ours.”

“It means a lot to stand alongside parents and caregivers in their pain, to lend a hand—even being there to help. I know this for a fact, there is nothing like someone’s presence and support in whatever way big or small in lightening the burden of a CHD patient. This also strengthened me.”

“Don’t only focus on our hearts but our whole body and mental health. These need to be ongoing conversations, not talked about once and forgotten.”

On the most basic level, patient organizations offer a unique tool for patients and caregivers to meet and share experiences, and these connections can be transformational. Many adults with CHD have never met anyone else with a similar condition, and most parents will not be connected to other families whose child needs open-heart surgery or is living with ongoing heart challenges. Peer connections have been shown to be effective in supporting the mental health of both caregivers and patients with CHD, and virtually all patient organizations provide patients and caregivers ways to support each other.^10^^,^^11^

The Internet has changed the way patients with CHD and their families connect. There are over 150 Facebook groups dedicated to patients with CHD and families, ranging in size from a few dozen to over 10,000 members, each serving as important resources for patients and their families searching for support and information. Patient organization–run forums are typically closely moderated to ensure that the conversation remains supportive, and that people refrain from providing medical advice. These groups are available 24/7 from the privacy of home and can be a lifeline for patients and families negotiating major challenges. Where Internet access is expensive or unreliable, such as Uganda and Namibia, WhatsApp is also widely used to connect patients and families. In countries such as India and Malaysia where social stigma is common, patient organizations report that many members will only interact virtually as disclosure of a CHD diagnosis can have a major negative impact on social interactions, marriage eligibility, and employment prospects. A major focus of these organizations is helping these patients, and their families overcome the shame and fear often associated with CHD through meeting others like themselves and connecting their members in a way that guarantees privacy.

“Being in Zipper Sisters Facebook group has helped me feel NOT alone in so many instances where I felt alone. Having people that understand not just me as a woman, but as a patient has been amazing!”

**Figure 2:**
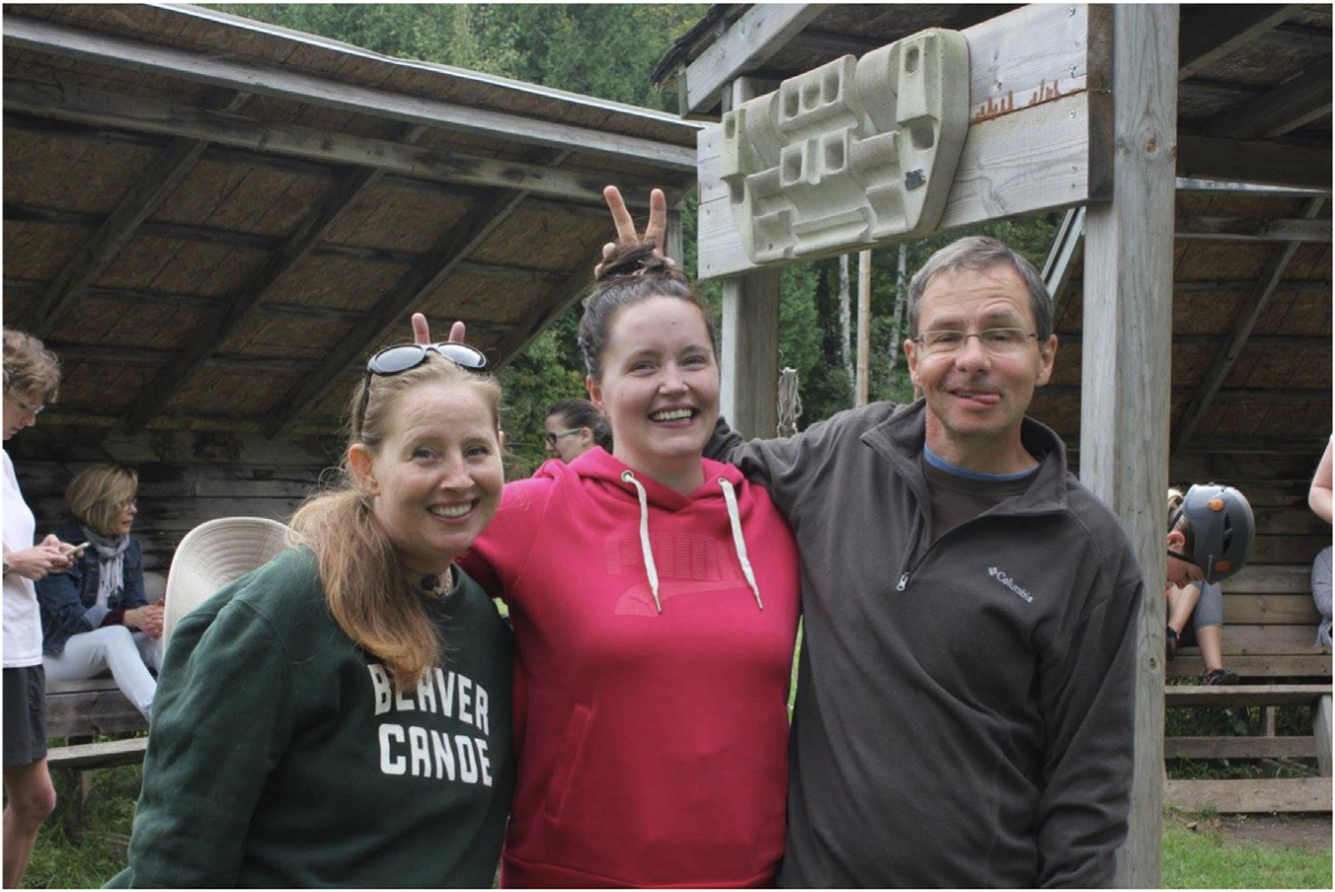
Campers at the Beat Retreat Camp, run by the Canadian Congenital Heart Alliance. Used with permission from the Canadian Congenital Heart Alliance and Beat Retreat Camp.

When possible, meeting in person deepens and broadens patient and family connections, and groups offer a wide variety of resources to encourage connection. For CHD patients and parents who are not interested in formal “support groups,” in-person educational evenings or social events such as holiday parties may be more appealing. Larger events, such as the awareness walks organized by organizations such as the Pakistan Children’s Heart Foundation^12^ and the Adult Congenital Heart Association,^13^ provide opportunities for people to meet and connect. Social activities can range from sauna parties in Finland^14^ to Flamenco nights in Spain,^15^ and many groups also bring CHD patients and families together to enjoy physical activities such as kayaking, hiking, and in Germany, swimming classes specifically for children with CHD.^16^ Attending overnight camps for children and adults, such as Canada’s Beat Retreat Camp^17^ (Fig. 2), offers an even richer opportunity to build trust and relationships between patients and their health care providers. Although some countries have privacy protections that can prohibit contact with patients in hospital, organizations such as Sweden’s Hjartbarnsfonden^18^ and Spain’s Menudos Corazones^19^ provide trained volunteers who offer inpatient support. In New Zealand,^20^ parent volunteers work directly with the hospital psychologists and social workers to provide comprehensive in-hospital support. Whichever way patients and families come together, interacting in person allows the development of bonds not available in virtual forums. When patients and families can actually see each other, they can compare scars, trade stories, and share hugs.

## Providing Educational Resources for Patients and Families

A large body of evidence documents major knowledge gaps among both patients with CHD and their caregivers, and these are often evident in the questions asked and misconceptions shared among patient and family communities.^21^, ^22^, ^23^, ^24^ Medical visits are often rushed, and care providers may lack time to elicit and answer the numerous questions that come with many clinical encounters. Patients and their families may feel intimidated in medical settings or avoid asking questions for fear of revealing how little they know. Although many clinicians strive to use simple language and provide clear information, there is often a large gap between “doctor-speak” and “patient-speak,” as well as language and cultural barriers.

Patient and family groups all over the world offer extensive educational resources to their members, in print material, on websites, webinars, and in-person education events. For example, the Canadian Congenital Heart Alliance^25^ has been a partner in many paediatric and adult CHD patient educational events across Canada, interacting with patients and their families and engaging in panels and workshops outside of the clinic setting. Many patient organizations run educational sessions on specific topics, such as pregnancy planning; this topic has been specifically covered by Canadian Fontan,^26^ the Adult Congenital Heart Association,^13^ Kenya Mended Hearts,^27^ and CHD Malaysia.^28^ Other topics include mental health issues, heart failure risk factors, heart valve replacement surgeries, and end of life care. These events provide patients a comfortable, supportive setting to ask questions and gather information, either in person or via online chat. A unique benefit of this type of event is that patients learn about their conditions without having to ask their clinician questions when they may not even know where to begin.

Patient organizations take their responsibility seriously, and as such ensure that educational resources, including webinars, handouts, articles, videos, and website content, are developed in partnership with clinicians and reviewed before being shared with the public. These partnerships are beneficial to clinicians too, by giving them a window into the issues that matter most to patients, knowing what resources are available to patients, and helping organizations provide their communities with up-to-date best-practices–based resources.

### Education through peer-to-peer interaction

Most patient education takes place in informal settings, such as online chat groups, where patients and their families ask questions of each other. Zipper Sisters: Women with CHD,^29^ for example, is a female CHD patient-only moderated Facebook group with thousands of members. The moderators and seasoned members work hard to ensure that accurate information, including links to resources, is shared and misinformation quickly removed. Peer-to-peer groups like this offer patients not only 24/7 support but also a community where they can ask questions that they may not feel comfortable asking their health care team.

### Education that can respond to real-time needs

Shortly after the start of the pandemic, although there was a growing body of medical information available on COVID-19 and heart disease, there was a real lack regarding the effects of COVID-19 on congenital heart patients. As a result, misinformation and confusion was rampant. In response, Global ARCH convened monthly meetings with patient family leaders to share information and discuss the challenges each country was facing. It also collaborated with the International Quality Improvement Collaborative for CHD, and medical experts, to create the *COVID-19 Fact Sheet for People with CHD and Rheumatic Heart Disease*.^30^ It is an easy-to-read document that was translated into 9 languages within a few weeks and widely shared via patient and family organizations and at numerous CHD surgical centres in low-resource countries. It gave member organizations a useful, easy-to-read, reliable resource to educate their communities about COVID, the risk factors for patients, and helped to clarify information about the COVID-19 vaccine. The document was adapted for use by the Canadian Congenital Heart Alliance, Heart & Stroke, and several other Canadian CHD organizations.

Many other groups created their own educational resources, including Conquering CHD,^31^ that provided weekly COVID-19 bulletins. These types of resources can play a critical role in patients’ lives, nimbly filling a gap that larger organizations and institutions fail to address.

### Education to help prevent loss to care

One area where many patient and family organizations continue to observe widespread misinformation and knowledge gaps is the need for lifelong care maintenance. As noted above, congenital cardiologists only interact with patients who are in congenital heart care. Despite the focus on care maintenance and the creation of transition activities and programmes, the large majority of patients continue to be lost to care^32^ with rates of successful transfer to ACHD care reported to be 25% in Canada,^33^ 22% in Europe,^34^ 12% in the United States,^35^ and less than 5% in Japan.^36^ Loss to care starts in childhood; a 2009 Canadian study reported that just under 30% of children with CHD had stopped seeing a cardiologist before age 12,^37^ and a more recent US study found that 47% of families left cardiac care before age 5.^38^

“My baby had open heart surgery 3 months ago and was cleared today and will never need another surgery!”

“I was told I was fixed and everyone around me thinks this.”

“I don’t have a heart defect. I had a complete correction, so I don’t have it anymore.”

“What percentage of people here have had ‘one and done surgery’?”

Every day, patient and family organizations encounter patients and families who perceive themselves or their child as “cured” after surgery. Some of this is wishful thinking—that their child is now fine and will have a normal life. The commonly used terms “total repair,” “complete repair,” and “total anatomic repair” may lead parents to believe that their child is “completely fixed,” and cardiologists and other health care providers often use the past tense when asking parents about their child’s diagnosis (ie, what *did* she have?). This can confirm their belief that, once surgery is complete, they no longer have a heart defect. After the stress of surgery, many parents are eager to get back to a “normal life” free of medical visits, and those who have experienced a significant loss of income may hesitate to spend limited resources on return visits for a child who seems completely healthy. This can result in families leaving care before their medical team has begun discussing long-term risks and why return visits are important.

1. Recommendations to Providers to Help Prevent Loss to Care
  • Avoid using past tense when discussing post-operative diagnoses, for example, *“What did she have?”*
  • Avoid using “complete repair,” “total repair,” “complete anatomic repair,” and other words suggesting cure
  • Tell parents that CHD surgery is not curative at the time of the initial diagnosis
  • Reiterate that CHD requires lifelong care at every follow-up visit
  • Identify the specific risks that make lifelong follow-up care necessary
  • Explain the importance of receiving adult care from special ACHD cardiologists
  • Teach transition-age patients and their families what ACHD care is and how to find it

To be effective, preventing loss to care demands clear and accurate family education on the lifelong risks of CHD, starting in infancy and continuing throughout childhood. When parents join in-person or online groups and express the belief that their child is “cured,” they usually encounter others whose experiences prove this to be untrue. Groups that include both children and adults, such as the Canadian Congenital Heart Alliance,^25^ the Finnish Association of Heart Children and Adults,^14^ and Conquering CHD,^31^ provide parents and children an excellent window into the unforeseen challenges associated with ACHD. Although information on long-term risks can be frightening, many parents report that meeting older children, teens, and adults with CHD who are thriving provides them with hope about their child’s future. This cross-age design also provides patients with CHD and their families one lifelong support system and ready role models that can help them prepare for life going forward.

In low- and middle-income countries, the lack of paediatric cardiology services, economic burden of travelling for care, and the stigma associated with CHD results in few children receiving regular postsurgical follow-up. Groups such as Brave Little Hearts Zimbabwe^39^ have started expanding their advocacy message beyond the need for surgical access to include the need for a paediatric cardiac care system that can provide ongoing follow-up. In Pakistan, the Pakistan Children’s Heart Foundation^12^ is working to identify effective strategies to educate low-literacy families on the need for follow-up and overcome the stigma that can prevent them from seeking such care.

### Education to help patients find needed care

“My cardiologist says he will send me to an ACHD specialist when I need it.”

“Can someone recommend a good cardiologist in Sioux City?”

**Figure 3:**
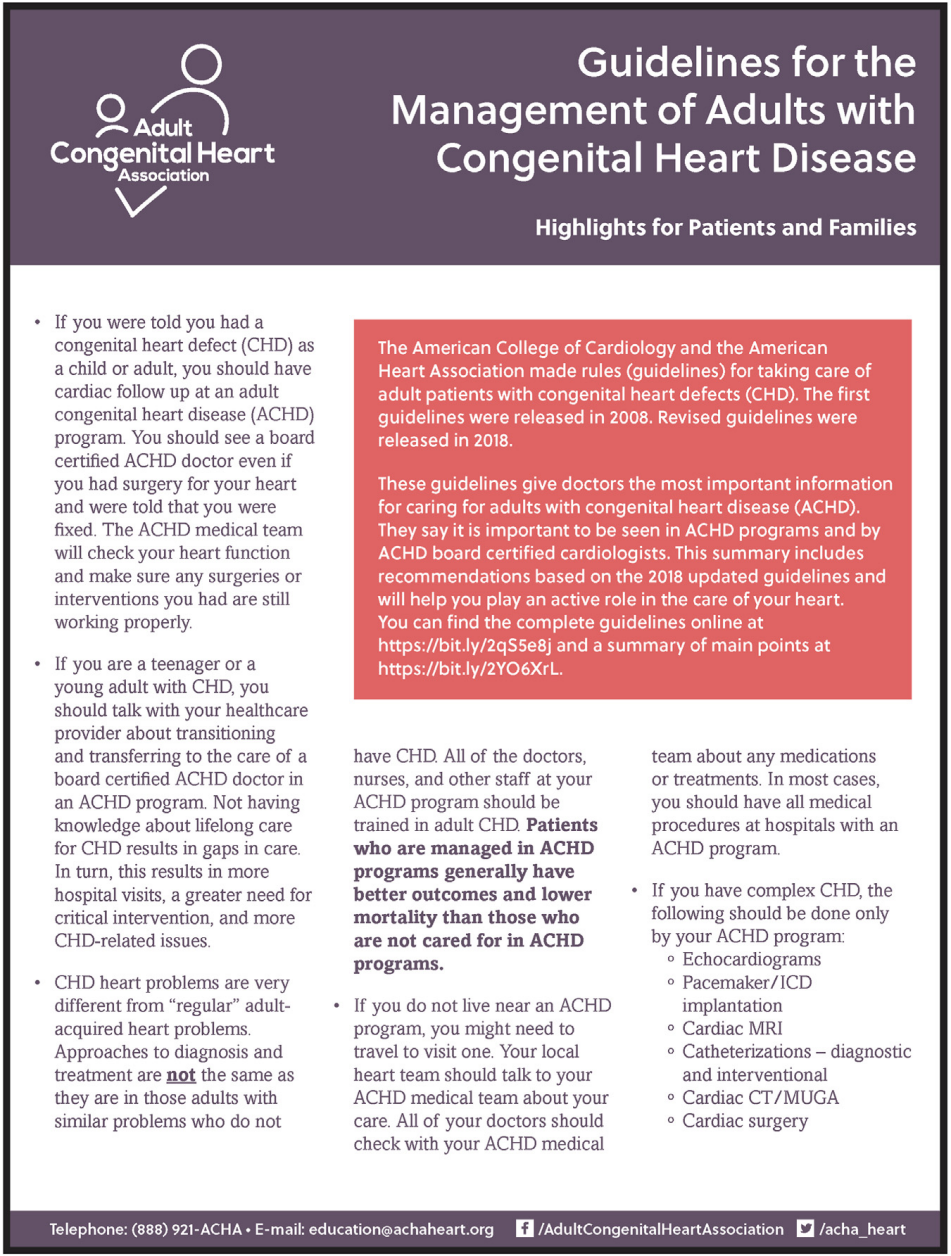
ACHA’s Guidelines for the Management of Adults with Congenital Heart Disease. Used with permission from the Adult Congenital Heart Association.

Many adults with CHD initiate contact with patient organizations in response to new health problems, thus providing an opportunity to educate them about their need for ACHD care. In the United States, the Adult Congenital Heart Association^13^ provides an easy-to-read summary of the AHA/ACC ACHD Care Guidelines on their website^40^ (Fig. 3), describing what an ACHD programme is and emphasizing that people seen at such programmes have a lower risk of premature death and disability than those seeing “regular” cardiologists.^41^ They also offer an online map of US ACHD centres and publish a directory of ACHD programmes available globally.

Even in countries such as Canada and the United Kingdom, whose national health systems have established networks of approved ACHD programmes, patients can and do choose to be seen elsewhere, as reflected in the data showing that fewer than half of European adults with CHD are receiving such care.^34^ Like the Adult Congenital Heart Association, the United Kingdom’s Somerville Heart Foundation^42^ and the Canadian Congenital Heart Alliance^25^ also have information about what ACHD care is and how to find it.

When finding a cardiologist, patients heavily rely on recommendations from friends, family, and Facebook friends, rather than guidelines or physician recommendations. This is where patient organization leaders and forum moderators can step in to guide patients to recommended care. For many patients and families, having members of their online support group tell them positive experiences about their own care team is the most powerful tool available to get them back to the specialized care they need.

Finally, patients need to understand the importance of seeking out cardiologists with special training in congenital cardiology. Although most transition programmes focus on supporting patients as they “graduate” to an ACHD programme preselected by their paediatric care team, this deprives the patient of the opportunity to become educated and empowered to make an independent, informed decision about where to seek care. This is a skill they will need every time they move to a different part of the country, or further afield, change insurance, and travel, or if they are unsatisfied with their current cardiologist.

“I would never tell my employer for fear of my condition being used against me. Such as if I was to try and gain job promotion.”

## Advocacy

Congenital heart patients cannot thrive without policies in place that ensure that they receive the high-quality, lifelong patient-centred care and social services essential to their health and well-being. Patient organizations around the world advocate for needed policy changes in a variety of arenas and work with professional associations, hospital systems, health departments, and lawmakers to ensure that their community’s needs are met.

### Global advocacy efforts

In many countries, including the United States and Mexico, campaigning efforts by parent organizations directly led to nation-wide infant pulse oximetry screening to detect CHD. In the United States, the Adult Congenital Heart Association,^13^ the Children’s Heart Foundation,^43^ and Mended Little Hearts®^44^ collaborated to draft the Congenital Heart Futures Act, proposed legislation mandating the expansion of government services to support the long-term well-being of patients with CHD. Patients and parents met directly with lawmakers and used their own personal stories to highlight the unmet needs of their community. The legislation passed in 2008 and led to a major expansion in government data collection aimed to identify gaps in services for the community with CHD. Ongoing advocacy has resulted in funding growing from CAD$1 million in 2009 to $5.5 million in 2021.

### Tackling social services to help patients

In addition to health care services, patients with CHD and families continue to lack social benefits such as disability support, educational services, access to low-cost insurance, and protection from workplace discrimination. In some settings, individuals with other forms of disease receive government services, but those with CHD are explicitly excluded. In others, patients with CHD are not formally excluded, but the mismatch between the application criteria and CHD health issues make qualifying virtually impossible. For example, heart disease–related questions may be designed for acquired heart disease, and reviewers may not have the expertise to evaluate CHD cardiac testing. In the United States, patient organizations brought CHD survivors together with government officials to provide in-person and written testimony on their challenges qualifying for disability benefits, resulting in the inclusion of complex CHDs on the list of qualifying conditions.^45^^,^^46^

In Spain, Menudos Corazones^19^ is also partnering with government to help make government benefits available to more patients with CHD and families who need them. In many countries, government health insurance ends when patients with CHD turn 18, leaving patients unable to access needed care. Corazones Luchadores^47^ is working to extend government insurance benefits in CHD past age 15, and patient organizations in Kerala, India, are urging the health ministry to expand funding to cover the growing population of adult CHD patients who survived thanks to free cardiac surgery but cannot access insurance after age 18.

### Advocating for mental health services

“Mental health assessments and support must be part of comprehensive care for all people with CHDs rather than a special service that is offered only in some places or in special circumstances.” *American Heart Association*^48^

Despite well-documented high rates of emotional and psychological challenges among patients with CHD globally,^49^^,^^50^ patient organizations around the world report that the psychological needs of the CHD community continue to be neglected. This is in marked contrast to other paediatric-onset diseases such as cancer, in which integrated psychological services, often led by trained psycho-oncologists, are a standard part of the health care team.^51^ Although a few countries have invested substantially in CHD psychological support, in most places, a shortage of hospital funding and staffing prevent adequate levels of service to be delivered. In the United States, the Adult Congenital Heart Association advocacy efforts ensured that the availability of social workers and/or psychologists was incorporated into the US adult CHD programme accreditation guidelines, but many centres report that they do not have funds available to meet this standard. In 2022, the American Heart Association published a scientific statement addressing the mental health needs of patients with CHD^48^ and acknowledged that despite decades of research documenting the acute mental health needs of patients and families, and ongoing advocacy efforts by patient organizations, little progress has been made in providing the mental health services essential for well-being. It calls for the integration of dedicated mental health professionals in all paediatric and adult congenital cardiac care teams and encourages patient organizations to continue to push for the adequate provision of mental health services for all patients and families living with CHD.

### Advocating for quality care

Every person born with CHD deserves the highest-possible level of care, and patient organizations strive to make this happen. For example, when it became clear that one set of children sent abroad for CHD surgery were dying at much higher rates than others, Kenya Mended Hearts successfully petitioned to stop government funding for the underperforming hospital. In the United States, Conquering CHD partnered with professional organizations to ensure that CHD surgical outcomes data were publicly available, presented in a way that was meaningful to patients, and included the determination of CHD hospital ratings. In the United Kingdom, the Somerville Heart Foundation^42^ worked with the National Health Service to develop an official ACHD care pathway and ensure that the services provided were patient-centred and met their community’s needs. In 2018, Bulgaria’s only children’s heart surgery hospital^52^ was threatened by a lack of funds and staff. Coming together via Facebook, over 1000 affected parents and patients signed an open letter asking the government not to execute proposed cuts. Next, they organized a protest to demand an increase in hospital funding and staffing. Through these tactics, their access to high-quality in-country care was preserved. In the United States, the Adult Congenital Heart Association initiated and runs the ACHA ACHD Accreditation Programme, which ensures that ACHD programmes offer the staffing, services, and quality care all patients deserve. The accreditation programme was officially launched in 2017, and there are now over 50 US accredited centres.

### Global ARCH: creating a common voice for the CHD community

Although CHD is the most common birth defect and a major cause of infant and child mortality and morbidity worldwide,^53^ many governments and policy makers have minimal awareness of its prevalence and impact. Although the vast majority of patients with CHD born in high-income countries now reach adulthood, 90% of the world’s children with CHD, born in low- and middle-income countries, will die due to lack of access to cardiac care. In high-income countries, government investment continues to lag when compared with other diseases; in the United States, there are close to 3 million children and adults living with CHD,^54^ yet this population receives a tiny percentage of the federal funding. For example, in 2023, the National Institutes of Health invested $143 million in research funding in CHD, which is present in 1 in 100 babies, whereas childhood leukemia, which affects 1 in 13,500 children, received $268 million.^55^^,^^56^

Public awareness of CHD continues to lag compared with other, much-less-common, childhood-onset diseases such as paediatric cancer and cystic fibrosis. In many cases, the levels of awareness and funding seen in other disease states are a direct result of advocacy by patient organizations on behalf of their community. In 2017, Global ARCH was founded to accelerate policy change by strengthening the collective voice of those impacted by CHD. Membership is free, and participating organizations are provided with ongoing training, peer support, and opportunities to engage in collective action.

**Figure 4:**
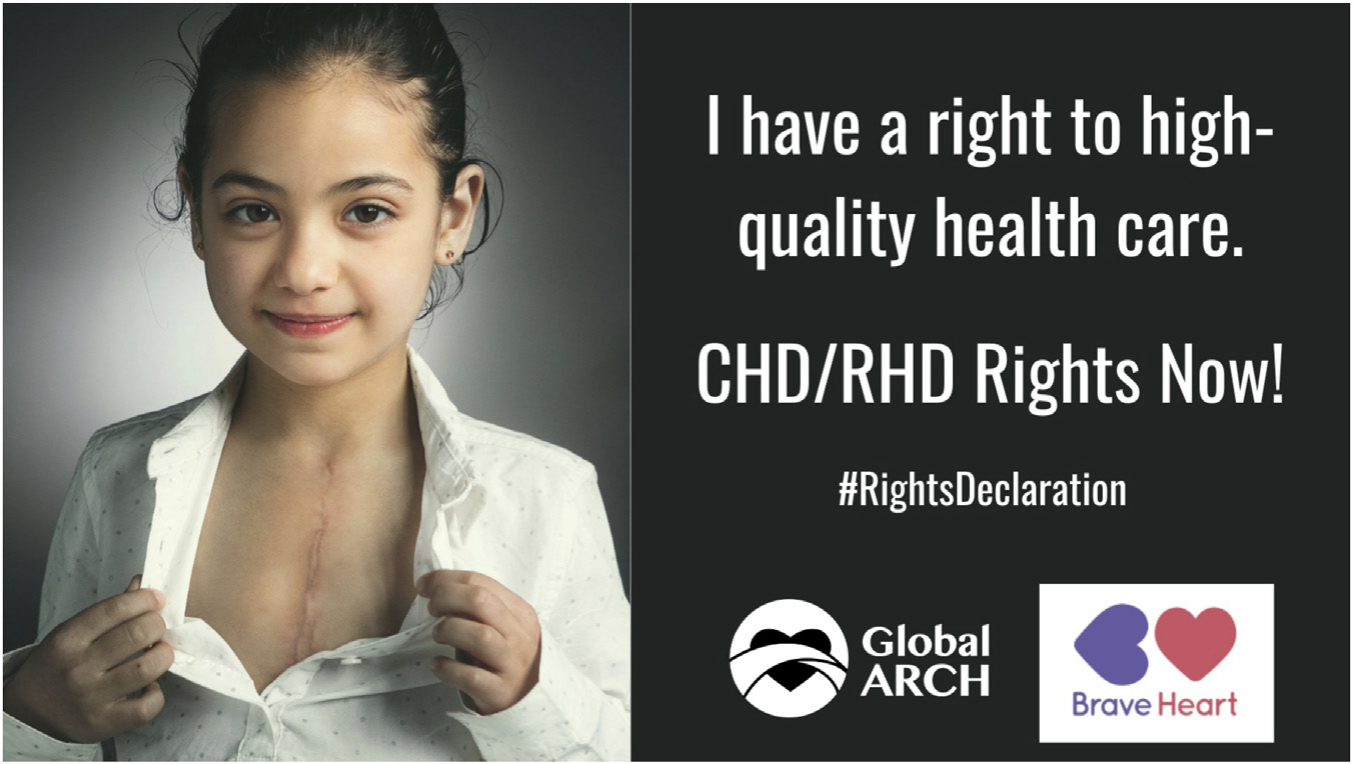
Declaration of Rights, developed by Global Alliance for Rheumatic & Congenital Heart Disease (ARCH). Used with permission from Global ARCH.

**Figure 5:**
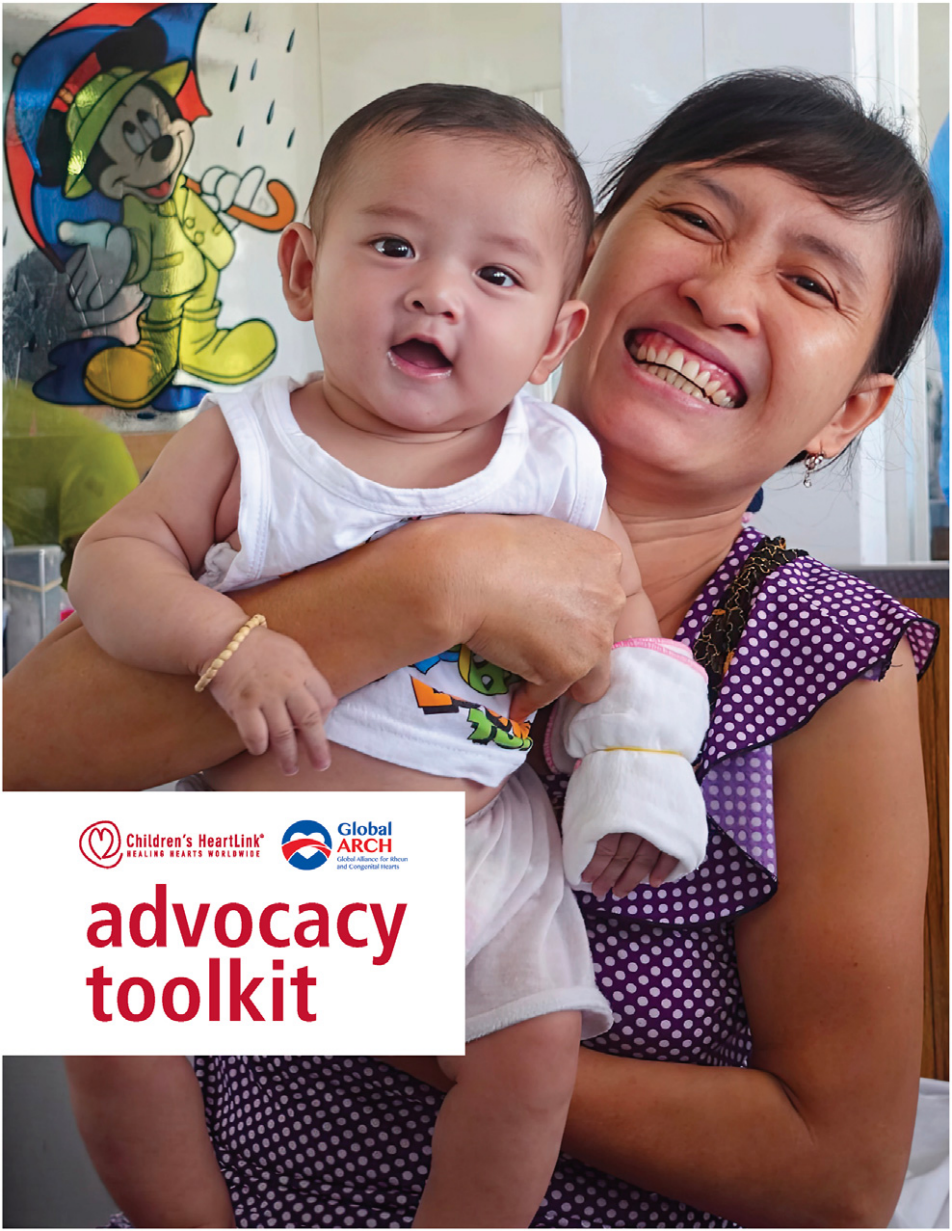
Global Alliance for Rheumatic & Congenital Heart Disease Advocacy Toolkit. Used with permission from Global ARCH.

In 2020, Global ARCH released the “Declaration of Rights for Individuals with Childhood-Onset Heart Disease”^57^ (Fig. 4), which states that every person with CHD has the right to the highest-possible health and well-being, and outlines specific actions that every government should take to ensure that patients with CHD achieve their right to health. In partnership with Children’s HeartLink,^58^ it has published an *Advocacy Toolkit*^59^ (Fig. 5), which offers information, resources, and practical tools to help patient organizations define advocacy goals and communicate them to different audiences. At the global level, Global ARCH works to bring patient with CHD and family representatives to venues such as the World Health Assembly, the UN General Assembly, and the World Heart Summit, and is working to heighten awareness of childhood-onset heart disease among global policy makers. It also brings its member organizations together to execute joint campaigns highlighting that lifelong CHD care is a human right.

“Global ARCH gives us a voice that can be heard by people around the world.”

## Conclusion

Over the past 20 years, CHD patient organizations have evolved from small support groups in paediatric cardiac wards to influential providers of education, support, resources, and advocacy for their communities. There has been a huge shift in patient knowledge and empowerment thanks in large part to patient organizations worldwide, making the patient experience less isolating and one of community, learning, and knowledge. It is a remarkable evolution, and one that can only help to improve the patient experience and ultimately patient outcomes. There are, however, major gaps that still need to be addressed, and one way forward is through partnerships between health care providers and patient organizations, and between patient organizations themselves, in research and education; advocating for standards, laws, and policies to support patients and their families; and joining global movements to improve patient survival and quality of life in underserved areas of the world.

2. Recommendations for Health Care Providers
  • Let patients know about patient support groups and organizations and encourage them to join
  • Become familiar with the resources provided by patient organizations and recommend them to patients and families
  • Partner with patient organizations on educational and supportive initiatives and activities
  • Advocate for mental health support for patients with CHD within your institution
  • Partner with patient organizations when conducting research to ensure that their priorities and perspectives are included in research design, and that they are involved in planning, execution, and dissemination
  • Identify potential leaders among your patients and encourage them to volunteer at their local organization

### Selected resources

• Canadian Congenital Heart Alliance (cchaforlife.org)
• Western Canadian Children’s Heart Network (https://wcchn.ca/)
• Children’s Heart Network of BC Society (www.childrensheartnetwork.org)
• Canadian Fontan (https://canadianfontan.com)
• FindACHD (@FindACHD on Twitter)
• Fondation en Cœur (Quebec) (https://en-coeur.org/)
• Braveheart Support Society (Nova Scotia)—Facebook
• Adult Congenital Heart Association (achaheart.org)
• Global Alliance for Rheumatic & Congenital Hearts (www.global-arch.org)
• Zipper Sisters—Facebook (private)
• The Heart Dialogues (Leigh Kamping-Carder—https://theheartdialogues.substack.com/)

### Camps

• Canadian Congenital Heart Alliance’s Beat Retreat (for adults with CHD)—connect via Facebook
• Camp Oki (Canadian camp for children with CHD—www.campoki.ca)
• Camp Braveheart (at Brigadoon Village in Nova Scotia—https://brigadoonvillage.org)
• Camp Odayin (US camp for children with CHD—https://campodayin.org)
• Camp del Corazon (camp for children and families living with congneital heart disease—https://www.campdelcorazon.org/)

## References

[1] Girouard H.S., Kovacs A.H. (2020). Congenital heart disease: education and employment considerations and outcomes. Int J Cardiol Congen Heart Dis.

[2] Chowdhury D., Johnson J.N., Baker-Smith C.M. (2021). Health care policy and congenital heart disease: 2020 focus on our 2030 future. J Am Heart Assoc.

[3] Biber S., Andonian C., Beckmann J. (2019). Current research status on the psychological situation of parents of children with congenital heart disease. Cardiovasc Diagn Ther.

[4] Kovacs A.H., Saidi A.S., Kuhl E.A. (2009). Depression and anxiety in adult congenital heart disease: predictors and prevalence. Int J Cardiol.

[5] WHO Nothing for us, without us: opportunities for meaningful engagement of people living with NCDs: meeting report. https://apps.who.int/iris/handle/10665/340737.

[6] Van Reusen A.K., Wehmeyer M.L., Sands D.J. (1998). Making It Happen: Student Involvement in Education Planning, Decision-Making and Instruction.

[7] Global Alliance for Rheumatic and Congenital Hearts. https://global-arch.org/.

[8] Lopez K.N., Morris S.A., Tejtel S.K.S., Espaillat A., Salemi J.L. (2020). US mortality due to congenital heart disease across the lifespan from 1999–2017 exposes persistent racial/ethnic disparities. Circulation.

[9] OECD. OECD Skills Outlook 2013: first results from the survey of adult skills. OECD Publishing; 2013. Available at: 10.1787/9789264204256-en. Accessed August 2, 2023.

[10] Carlsson T., Klarare A., Mattson E. (2020). Peer support among parents of children with congenital heart defects: a qualitative analysis of written responses submitted via an online survey. J Adv Nurs.

[11] Callus E., Pravettoni G. (2018). The role of clinical psychology and peer to peer support in the management of chronic medical conditions—a practical example with adults with congenital heart disease. Front Psychol.

[12] Pakistan Children’s Heart Foundation. https://pchf.org.pk/.

[13] Adult Congenital Heart Association. https://www.achaheart.org/.

[14] Sydanlapset ja-aikuiset. https://sydanlapsetjaaikuiset.fi/.

[15] Menudos Corazones. https://www.menudoscorazones.org/agenda.

[16] Bunderverband Herzkranke Kinder e.V. https://bvhk.de/.

[17] Facebook. The beat retreat. https://www.facebook.com/groups/125717150861456.

[18] Hjarte Barns Fonden. https://hjartebarnsfonden.se/.

[19] Menudos Corazones. https://www.menudoscorazones.org/.

[20] Heart Kids N.Z. Once a heart kid, always a heart kid. https://www.heartkids.org.nz/.

[21] Lesch W., Specht K., Lux A. (2014). Disease-specific knowledge and information preferences of young patients with congenital heart disease. Cardiol Young.

[22] Gramszlo C, Karpyn A, Christofferson J, et al. Meeting parents’ needs for education and preparation following congenital heart disease diagnosis: recommendations from a crowdsourced study [e-pub ahead of print]. Am J Perinatol. doi:10.1055/a-1906-8786, accessed September 12, 2022. 10.1055/a-1906-8786 PMC10008463 35863373

[23] Cheuk D.K.L., Wong S.M.Y., Choi Y.P., Chau A.K.T., Cheung Y.F. (2004). Parents’ understanding of their child’s congenital heart disease. Heart.

[24] Moons P., De Volder E., Budts W. (2001). What do adult patients with congenital heart disease know about their disease, treatment, and prevention of complications? A call for structured patient education. Heart.

[25] Canadian Congenital Heart Alliance. https://www.cchaforlife.org/.

[26] Canadian Fontan Connection. https://canadianfontan.com/.

[27] Kenya Mended Hearts Patient’s Association. https://kenyamendedhearts.com/.

[28] Facebook. C.H.D Malaysia. https://www.facebook.com/profile.php?id=100079309161358.

[29] Facebook. Zipper sisters: women with CHD. https://www.facebook.com/groups/121190384672035.

[30] Global Arch. CHD and COVID-19. https://global-arch.org/learn-more/chd-and-covid-19/.

[31] Conquering CHD https://www.conqueringchd.org/.

[32] Thakkar A.N., Chinnadurai P., Huie Lin C. (2017). Adult congenital heart disease: magnitude of the problem. Curr Opin Cardiol.

[33] Moons P., Skogby S., Bratt E.-L. (2021). Discontinuity of cardiac follow-up in young people with congenital heart disease transitioning to adulthood: a systematic review and meta-analysis. J Am Heart Assoc.

[34] Thomet C., Moons P., Budts W. (2019). Staffing, activities, and infrastructure in 96 specialised adult congenital heart disease clinics in Europe. Int J Cardiol.

[35] Gerardin J., Raskind-Hood C., Rodriguez F.H. (2019). Lost in the system? Transfer to adult congenital heart disease care—challenges and solutions. Congenit Heart Dis.

[36] Ochiai R., Kato H., Akiyama N. (2016). Nationwide survey of the transfer of adults with congenital heart disease from pediatric cardiology departments to adult congenital heart disease centers in Japan. Circ J.

[37] Mackie A., Ionescu-Ittu R., Therrien J. (2009). children and adults with congenital heart disease lost to follow-up. Who and when?. Circulation.

[38] Jackson J.L., Morack J., Harris M. (2019). Racial disparities in clinic follow-up early in life among survivors of congenital heart disease. Congenit Heart Dis.

[39] Facebook Brave Little Hearts Zimbabwe. https://www.facebook.com/bravelittleheartszim/.

[40] ACHD. ACHD care guidelines. https://www.achaheart.org/your-heart/resources/achd-care-guidelines/?gad=1&gclid=CjwKCAjw-7OlBhB8EiwAnoOEk1DHZjcBzC0h1SJMAZ4GdrPxtWx_ZMmybtkgfuu8e33nrJ1t8ViGHxoCgscQAvD_BwE.

[41] Diller G.P., Orwat S., Lammers A.E. (2021). Lack of specialist care is associated with increased morbidity and mortality in adult congenital heart disease: a population-based study. Eur Heart J.

[42] Somerville Heart Foundation. https://sfhearts.org.uk/.

[43] The Children’s Heart Foundation. https://www.childrensheartfoundation.org/.

[44] The Mended Hearts Inc. https://mendedhearts.org/.

[45] Disability Benefits Center Compassionate allowance—single ventricle. https://www.disabilitybenefitscenter.org/compassionate-allowances/single-ventricle-social-security-disability.

[46] Social Security Program Operations Manual System (POMS). https://secure.ssa.gov/poms.nsf/lnx/0423022590.

[47] Facebook Fundación Corazones Luchadores Chile. https://www.facebook.com/CorazonesLuchadoresChile/.

[48] Kovacs A., Brouilette J., Ibeziako P. (2022). Psychological outcomes and interventions for individuals with congenital heart disease: a scientific statement from the American Heart Association. Circ Cardiovasc Qual Outcomes.

[49] Westhoff-Bleck M., Briest J., Fraccarollo D. (2016). Mental disorders in adults with congenital heart disease: unmet needs and impact on quality of life. J Affect Disord.

[50] Kolaitis G.A., Meentken M.G., Utens E.M.W.J. (2017). Mental health problems in parents of children with congenital heart disease. Front Pediatr.

[51] Mavrides N., Pao M. (2014). Updates in pediatric psycho-oncology. Int Rev Psychiatry.

[52] Child's Heart Association. https://childsheart.eu/.

[53] Zimmerman M., Smith A., Sable C.A. (2017). Relative impact of congenital heart disease on morbidity and mortality in infancy around the globe: the global burden of disease study. Circulation.

[54] CDC Data and statistics on congenital heart defects. https://www.cdc.gov/ncbddd/heartdefects/data.html.

[55] NIH Estimates of funding for various research, condition, and disease categories (RCDC). https://report.nih.gov/funding/categorical-spending#/.

[56] Leukemia and Lymphoma Society Childhood and adolescent blood cancer facts and statistics. https://www.lls.org/facts-and-statistics/childhood-and-adolescent-blood-cancer-facts-and-statistics#:%7E:text=The%20age%2Dadjusted%20incidence%20rate,4.9%20and%20lymphoma%2C%202.5.

[57] Global Arch Declaration of the rights of individuals affected by childhood-onset heart disease. https://global-arch.org/wp-content/uploads/2022/02/FinalDeclarationRightsCHDRHD_web.pdf.

[58] Children’s HeartLink. https://childrensheartlink.org/.

[59] Global Arch. Advocacy toolkit. https://global-arch.org/wp-content/uploads/2023/02/Advocacy-Toolkit_2023.pdf.

